# Interpretable driver behavioral state monitoring using facial landmarks for human-autonomous vehicle interaction

**DOI:** 10.3389/frobt.2026.1896859

**Published:** 2026-10-23

**Authors:** Milton O. Candela, Jesus A. Mandujano-Granillo, Ismael Vidal-Lopez, Mauricio A. Ramírez-Moreno, Juan C. Tudon-Martinez, Jorge De-J. Lozoya-Santos

**Affiliations:** Tecnologico de Monterrey, School of Engineering and Sciences, Monterrey, Mexico

**Keywords:** autonomous vehicles, driver monitoring system, facial features, human machine interface, machine learning, facial landmarks, deep learning, support vector machines

## Abstract

**Introduction:**

Behavior-aware robotic systems require both accurate human state recognition and transparent machine intent to support trust and safe collaboration. This study presents an integrated framework for autonomous vehicles combining a real-time vehicle state visualization interface with a calibration-driven facial-based behavioral state recognition module.

**Methods:**

A Polaris GEM e6 autonomous vehicle equipped with drive-by-wire control and LiDAR served as the testbed. A real-time Human-Machine Interface (HMI) built with ROS and Foxglove Studio visualized localization, object detection, and driving states. Concurrently, a vision-based behavioral monitoring system used an Intel RealSense L515 camera, MediaPipe, and OpenCV to extract facial landmarks. Individual calibration established neutral baselines for eye aspect ratio, mouth aspect ration, and head rotation angles. A real-time threshold-based finite-state model facilitated data collection across five behavioral states: neutral, closed eyes, out-of-safe-zone, yawning, and fast blinking. Classical classifiers (Support Vector Machine [SVM], Random Forest [RF]), and deep learning architectures (Convolutional Neural Networks, Recurrent Neural Networks) were trained on a controlled indoor dataset using stratified k-fold cross-validation (k = 10), and evaluated on a hold-out outdoor dataset.

**Results:**

The HMI provided stable, real-time visualization of vehicle state and perception, while the facial monitoring system achieved a ∼ 98.5% face detection success rate. The online heuristic model identified behavioral states and enabled high-quality data collection, capturing features with strong Kruskal-Wallis significance from the neutral state for closed eyes (EAR, p<0.001 ), yawning (MAR, p<0.001 ), fast blinking (blink rate, p<0.01 ), though out-of-safe-zone was non-significant (head rotation, p>0.05 ). Stratified cross-validation accuracy and F1-scores reached ∼ 91% for SVM and RF. On the outdoor test data, SVM maintained its performance, whereas RF dropped to a ∼ 70% F1-score due to inter-session variability and feature ambiguity. Deep learning architectures achieved test F1-scores ( ∼ 80%), comparable to the heuristic baseline model ( ∼ 81%). SVM was the most stable and best performing classifier, isolating its confusion to the out-of-safe-zone and neutral states.

**Conclusion:**

Combining transparent interface design with personalized calibration supports human-aware autonomy. The machine learning framework provided interpretable monitoring, while real-time visualization enhanced situational awareness, together contributing to behaviorally responsive and transparent human-robot interaction platforms.

## 1 Introduction

Autonomous vehicle (AV) technology has progressed rapidly in recent years, offering the potential to improve mobility, reduce traffic congestion, and enhance road safety. Despite these advances, widespread adoption remains limited, largely due to challenges in user trust and acceptance (Ekman et al., 2018; Martínez-Díaz and Soriguera, 2018). As vehicles assume greater levels of automation, the role of the human shifts from active driver to supervisory user. This transition introduces new requirements for human-autonomous system interaction, where users must understand system behavior while the system must interpret human states and intentions (Wilson et al., 2022).

A major source of uncertainty in automated systems arises from limited transparency in decision-making and communication (Davoli et al., 2020; Ekman et al., 2018). Even when autonomous vehicles possess sophisticated perception and planning capabilities, users often receive minimal information about the vehicle’s internal reasoning, environmental awareness, or intended actions (Ge et al., 2018). This lack of intuitive interaction can lead to confusion, discomfort, and reduced trust, particularly in scenarios requiring shared control or human takeover (Wang et al., 2022). Detecting user states such as attention, stress, fatigue, or disengagement is therefore critical for ensuring safe interaction and effective takeover readiness (Li et al., 2020; Zohaib et al., 2024).

Within autonomous vehicles, Driver Monitoring Systems (DMS) have emerged as a key mechanism for assessing user takeover readiness, engagement, and well-being (Davoli et al., 2020; Mehmood et al., 2024; Nazira et al., 2021; Wilson et al., 2022). Monitoring the driver’s cognitive and behavioral state is particularly important in partially automated driving scenarios, where humans may still need to intervene during unexpected events (Martínez-Díaz and Soriguera, 2018). Traditional monitoring approaches have explored physiological signals such as Electroencephalography (EEG) (Imran et al., 2024; Ramírez-Moreno et al., 2021; Zhu et al., 2021), Electrocardiography (ECG) (Cos et al., 2023; Imran et al., 2024; Kawanaka et al., 2013), Heart Rate Variability (HRV) and Electrodermal Activity (EDA) (Arakawa, 2021; Cos et al., 2023; Nisa’ Minhad et al., 2016), and speech analysis (Deng et al., 2024). While these signals provide valuable insights into stress, workload, or fatigue, they often require specialized sensors that may be intrusive or impractical for widespread deployment in consumer vehicles (Mehmood et al., 2024; Prabhakar and Biswas, 2021; Ramírez-Moreno et al., 2026).

Among the available modalities, facial feature analysis has become one of the most practical and scalable approaches for behavioral state recognition in in-vehicle environments (Davoli et al., 2020; Li et al., 2020; Mehmood et al., 2022; Shafaei et al., 2019). Camera-based systems can continuously monitor facial expressions, eye movement, and head orientation using non-intrusive sensors already present in many modern vehicles (Shafaei et al., 2019). Features such as Eye Aspect Ratio (EAR), Mouth Aspect Ratio (MAR), gaze direction, and head pose can provide indicators of attention, fatigue, distraction, and emotional state (Kawanaka et al., 2013; Li et al., 2020; Mehmood et al., 2024; Vetturi et al., 2020). By analyzing these signals, autonomous systems can infer user engagement and adapt interaction strategies accordingly (Davoli et al., 2020; Zhu et al., 2021).

However, a key limitation of many behavioral state recognition systems lies in their reliance on complex Machine Learning (ML) models that operate as black boxes (Li et al., 2020; Zhu et al., 2021). In safety-critical domains such as autonomous driving, it is essential that automated decisions remain interpretable and explainable, enabling researchers, system designers, and users to understand how behavioral states are inferred from biometric signals (Davoli et al., 2020; García-Hernández et al., 2024). Interpretable models can improve system validation, facilitate debugging, and strengthen user trust by providing transparent reasoning behind automated assessments (Imran et al., 2024; Shafaei et al., 2019).

Motivated by these challenges, this work proposes an interpretable facial-based behavioral state recognition framework for human-autonomous vehicle interaction. The proposed approach uses non-intrusive facial features (EAR, MAR, head rotation, blink rate) to characterize driver behavioral states. These features were extracted using computer vision techniques and analyzed through supervised ML models capable of distinguishing between user states associated with attention, fatigue, and distraction. The system is implemented and evaluated within a controlled experimental setup using an autonomous vehicle platform equipped with an in-cabin camera. A structured calibration and validation protocol is used to generate labeled behavioral data, enabling the training and evaluation of interpretable classification models. By focusing on feature-based inference, the proposed framework aims to support behavior-aware human-autonomous vehicle interaction while maintaining interpretability and reliability in safety-critical environments.

## 2 Materials and methods

### 2.1 Experimental platform

The experimental platforms consists on a Neighborhood Electric Vehicle (NEV) (Mandujano-Granillo et al., 2024; Mellado-Reyes et al., 2025), a research-oriented autonomous platform developed by AutonomousStuff (Morton, IL, United States) and based on a modified Polaris GEM e6 electric shuttle (Figure 1A). The vehicle has been upgraded for full drive-by-wire operation via PACMod, enabling software-level control of steering, braking, acceleration, and gear shifting.

**FIGURE 1:**
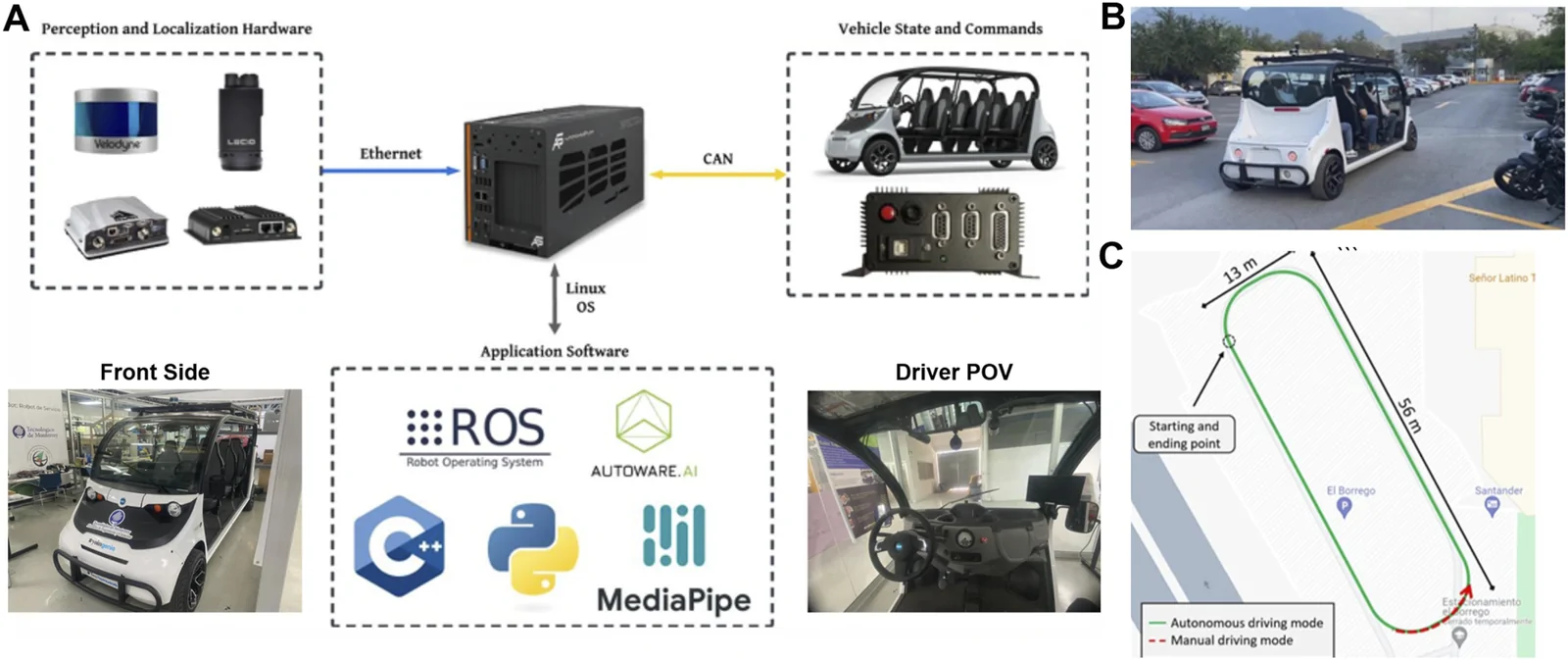
Experimental NEV platform for AV testing. **(A)** The Neighborhood Electric Vehicle (NEV) was upgraded for full drive-by-wire autonomous operation through (1) Perception and localization hardware (VLP-16 LiDAR, 3.2 MP cameras, and GNSS-IMU), (2) Vehicle State and Commands (PACMod), and (3) Application Software (ROS, Autoware. ai, C++, Python, Mediapipe). **(B)** NEV performing autonomous driving through a parking lot. **(C)** Parking lot map and route drove by the NEV platform.

The platform integrates environment perception and vehicle localization sensors, including dual Velodyne (San Jose, CA, United States) VLP-16 LiDARs, forward-facing Triton (Burnaby, Canada) 3.2 MP cameras, and a NovAtel (Calgary, Canada) GNSS-IMU system for real-time state estimation. All sensing, planning, and control processes are executed on an onboard Spectra industrial computer running Ubuntu and managed through a ROS-based modular architecture. Autonomous functionality is supported by the Autoware. ai software stack, providing localization, perception, and path-planning capabilities. Within this architecture, the NEV serves as a unified experimental testbed for both system-level interaction and user-state monitoring research (Mellado-Reyes et al., 2025), offering a robust environment for Human Machine Interface (HMI) development and facial feature-based monitoring, as well as real-time sensor integration and autonomous vehicle experimentation.

The objective of this HMI was to design and demonstrate an interface capable of clearly communicating vehicle state, motion intent, and environmental awareness to the user, by leveraging real-time access to vehicle speed, driving mode, localization, perception outputs (LiDAR and camera streams), and system status information. Validation focused on confirming reliable data extraction, processing, and visualization during autonomous operation. Formal, user-centered evaluation of the HMI was outside the scope of this validation phase, which focused on system level feasibility and real-time data integration.

The perception process followed the ROS-1 based Autoware. ai pipeline (Kato et al., 2018) in which raw LiDAR point cloud data undergoes a set of processes, such as ground segmentation, Euclidean clustering, shape estimation, and multi-object tracking to produce obstacle representations displayed by the HMI. To qualify the system’s responsiveness, sensor-to-topic latency was assessed offline from recorder ROS bag data by comparing each message’s header timestamp against its corresponding bag record time. For the LiDAR perception chain, by tracing each of its stages processing time across their nodes. Camera, GNSS, and DBW feedback topics, which include steering, velocity, braking, and gear state, exhibited 5 ms mean latency between sensor capture, and data availability on the ROS network. The LiDAR perception pipeline had a median end-to-end latency of approximately 78 ms from raw point cloud data acquisition to object tracking output. The HMI was run as Foxglove Studio on the same onboard computer as the ROS 1 master connected directly to the local ROS network, expecting minimal transport latency between data availability and panel update of less than 2 ms driven by frame-rendering time within the visualization client, which was not independently instrumented, representing a minor residual component not captured in this analysis.

Positioning accuracy was assessed at two levels. Path-tracking performance, which was computed with the lateral deviation between the vehicle’s estimated pose and the planned waypoint trajectory (Paden et al., 2016), showed a mean absolute error of 0.97 m, with an RMS of 1.06 m, and a 95th percentile of 1.67 m. Independently, the accuracy of the GNSS-IMU position source was assessed from the NovAtel OEM7 receiver’s reported position standard deviations (NovAtel Inc., 2026), yielding a mean spherical position error of 5.23 m. Throughout the recorded session, the receiver operated in a single point GNSS mode aided by an Inertial Navigation System (INS), consistent with standard GNSS/INS integration approaches used in vehicle navigation (Boguspayev et al., 2023), without active differential or Real-time Kinematic (RTK) corrections, which accounts for the large positioning errors relative to the smaller path-tracking error. The vehicle’s pose estimate use for control and path-tracking evaluation is the INS-fused output of the onboard GNSS-IMU system rather than the instantaneous raw GNSS fix, and IMU-aided smoothing between GNSS updates gives a pose estimate with lower noise for tracking purposes even though the GNSS correction quality remains unchanged.

Experimental validation was conducted in a closed parking area configured as a small oval track on campus (Figures 1B,C). This controlled environment enabled safe testing of autonomous navigation while verifying real-time system integration and visualization performance. The emphasis was on feasibility and system robustness rather than large-scale route evaluation or formal user studies.

### 2.2 Facial feature-based driver monitoring system

The facial feature-based Driver Monitoring System (DMS) is a non-intrusive, vision-based framework designed to assess driver attention and alertness using computer vision, face landmark detection, and ML techniques. It was implemented within the stationary in-cabin environment of the NEV autonomous vehicle platform. Data collection (N = 1) was conducted with the vehicle parked to ensure controlled, repeatable facial data acquisition.

An Intel RealSense L515 (Santa Clara, CA, United States) depth camera was mounted on the rear-view mirror to capture high-resolution facial video (Figure 2C). All processing was executed on the vehicle’s onboard Spectra computer running Ubuntu 20.04, ensuring full integration with the vehicle architecture. The implementation was developed in Python 3.8 using OpenCV for video acquisition and visualization, and MediaPipe Face Mesh for facial landmark extraction. The framework provides 468 three-dimensional landmarks per frame (Figure 2A), enabling computation of behavioral metrics without wearable sensors (Florez et al., 2023).

**FIGURE 2:**
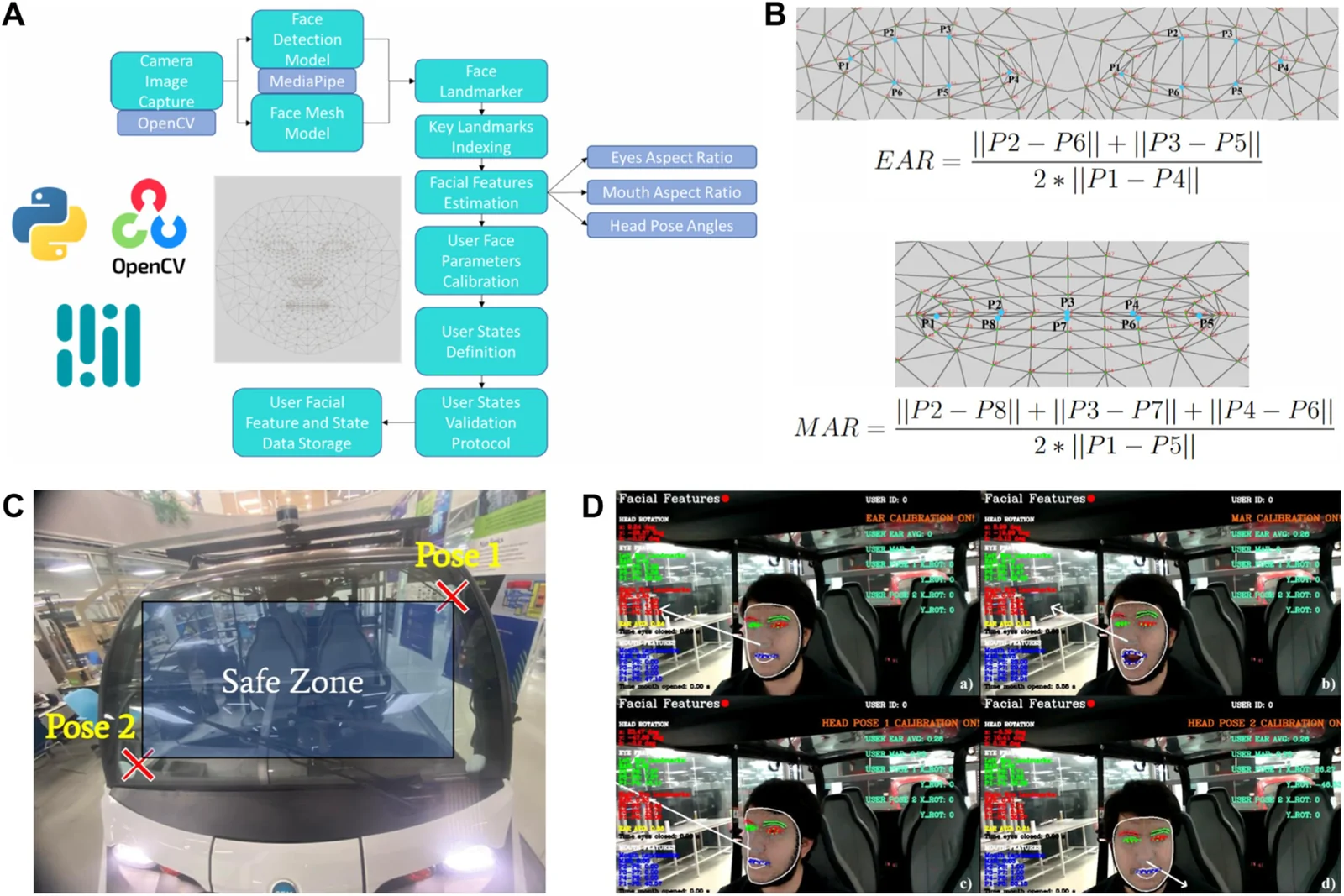
Facial features detection and extraction. **(A)** Python-based pipeline for face detection (OpenCV), facial keypoints and mesh model extraction (Mediapipe), landmark characterization and features estimation (EAR, MAR, head pose angles). **(B)** EAR and MAR features calculation based on Mediapipe mesh model and detected facial keypoints. **(C)** NEV “safe zone” defined by the two corners of the windshield to record yaw and pitch head angles. **(D)** Structured calibration protocol for a) EAR, b) MAR, c) head pose to first corner, and d) head pose to second corner.

Three primary indicators were extracted for their relevance to common driver behavior patterns, including drowsiness, distraction, and alertness (Biswal et al., 2021): (1) Eye Aspect Ratio (EAR) for eye closure detection (Chandiwala and Agarwal, 2021), (2) Mouth Aspect Ratio (MAR) for yawning detection (Gupta et al., 2018), (3) Head pose estimation (yaw and pitch) using OpenCV’s Perspective-n-Point (PnP) algorithm (Silic et al., 2024); EAR Equation 1 and MAR Equation 2 were calculated using selected landmark subsets defining contours of eye (left: [33, 160, 158, 133, 153, 144], right: [362, 385, 387, 263, 373, 380]) and mouth ([78, 81, 13, 311, 308, 402, 14, 178]), respectively (Figure 2B). Head orientation was estimated by mapping 2D facial landmarks (Sanil et al., 2023) (nose tip: 1, outer eye corners: [33, 263], mouth corners: [61, 291], chin: 199) to a canonical 3D facial model through the PnP algorithm, allowing real-time computation of gaze direction relative to a predefined safe zone (Figure 2C) (Silic et al., 2024).

$$\text{EAR}=\frac{\left\|P2-P6\|+\|P3-P5\right\|}{2*\|P1-P4\|}$$ (1)

$$\text{MAR}=\frac{\left\|P2-P8\|+\|P3-P7\|+\|P4-P6\right\|}{2*\|P1-P5\|}$$ (2)

### 2.3 Experimental validation

All computed metrics were recorded for offline analysis and behavioral classification considering a calibration, training, and testing protocol was implemented to account for intra-subject variability. For the calibration, the participant performed controlled facial actions to establish personalized baseline thresholds for EAR, MAR, and head rotation angles (Figure 2D). These included maintaining a neutral gaze for ${\text{EAR}}_{c}$ , opening the mouth in a relaxed state for ${\text{MAR}}_{c}$ , and orienting the head toward predefined corners of the windshield to record yaw and pitch angles that define a “safe zone” for gaze direction (Figure 2C) (Kim et al., 2019). The system recorded these values continuously and stored their average as baseline thresholds linked to the user’s unique identifier.

Once calibration was complete, the training phase started (Figure 2D). The user was guided through a sequence of actions that represent different behavioral states, including: (1) Neutral, (2) eye closure, (3) looking outside the safe zone, (4) yawning, (5) frequent blinking. These actions were performed over timed intervals and separated by short pauses to allow for reset. The system measured and saved facial feature values in real-time, while a threshold-based finite-state model determined the active state according to Table 1. The training phase consisted in the sequence:

1. State 0 - Neutral (0–15 s): The user begins by sitting naturally, facing forward, allowing the system to establish a baseline of the user’s normal, relaxed state.
2. State 0 - Intermission (15–25 s): A 10-s pause is provided as a buffer for the user to prepare for the next phase.
3. State 1 - Eyes Closed (25–40 s): The user is instructed to close their eyes for 15 s, testing the system’s ability to detect prolonged eye closure, critical for assessing driver alertness. This state’s baseline was the user’s calibrated EAR $\left({\text{EAR}}_{c}\right)$ , and State 1 was identified if the system detected ≤ 50% during ≥ 3 s. These thresholds were set given that an EAR between 0.18 and 0.25 determined whether eyes were closed (Dewi et al., 2022), and 3 s duration could detect a possible drowsiness state (Suryowinoto et al., 2024).
4. State 0 - Intermission (40–50 s): A 10-s pause for the user to reset and readjust.
5. State 2 - Look Outside Safe Zone (50–65 s): The user is asked to look outside the predefined “safe zone” of head rotation, which is defined by the calibration head angles. State 2 is identified when these angle limits are surpassed (Kim et al., 2019), hence, delimiting a line of sight based on the user’s head angles.
6. State 0 - Intermission (65–75 s): A 10-s intermission for the user to return to a neutral position.
7. State 3 - Yawning (75–90 s): The user simulates a yawn by opening their mouth, allowing the system to test its response to changes in the MAR, the threshold is set to > 30% ${\text{MAR}}_{c}$ . It was set given that maximum open mouth MAR values were between 0.5–0.9 (Albadawi et al., 2023).
8. State 0 - Intermission (90–100 s): A final 10-s pause before the last phase.
9. State 4 - Repeated Blinking (100–115 s): The user is asked to blink repeatedly, this EAR value is measured every 5 s due to the duration of the phase, with the blink duration baseline set to 0.4 s, and its threshold rate set to 30 blinks per minute. These threshold were set given that blink duration can vary between 100 and 400 ms (Albadawi et al., 2023), and that blink rate can vary between 10 and 25 blinks per minute at resting conditions (Maffei and Angrilli, 2018).

**TABLE 1:** User state definitions and threshold criteria.

State	Description	Detection variable	Threshold logic	Duration condition
0	Neutral	None	Reference state	None
1	Closed eyes	EAR	<50% ${\text{EAR}}_{c}$	≥ 3 s
2	Out of safe zone	Head rotation	$\theta \left(t\right)\notin \left[\theta_{\min},\theta_{\max}\right]$	Continuous
3	Yawning	MAR	>130% ${\text{MAR}}_{c}$	Continuous
4	Fast blinking	Blink rate	>30 blinks per min	≥ 0.4 s per blink

States were identified considering: (0) Reference MAR, EAR, and blink rate values of naturally sitting, relaxed, and facing forward, (1) less than 50% calibration EAR $\left({\text{EAR}}_{c}\right)$ for more than 3 s, (2) head rotation XY (θ) angle within the calibrated minimum $\left(\theta_{\min}\right)$ and maximum ( $\theta_{\max}$ ) “safe zone” angles, (3) more than 130% calibration MAR $\left({\text{MAR}}_{c}\right)$ , (4) more than 30 blinks per minute with more than 0.4 s per blink.

The protocol not only allowed for the real-time evaluation of the system’s responsiveness through the threshold-based finite-state model, but also provided labeled data on a frame-by-frame basis for offline analysis. By comparing detected and predicted states, the system’s classification performance was assessed to further refine its behavioral models. This protocol was replicated on an outdoor environment to build a hold-out testing dataset and evaluate model’s performance using the same calibration values, and training the models on the indoor training dataset.

### 2.4 Machine learning modeling and inference

Following the calibration and training procedure at the controlled environment using the NEV platform (Figure 1A), the facial feature dataset was further used for offline behavioral state classification. Thus, we developed predictive models that inferred behavioral user states from variations in facial biometric indicators including EAR, MAR, head rotation angles, and blink rate. Each time-labeled segment from the training routine was processed to extract statistical descriptors such as mean values and temporal variations of the recorded facial metrics. These features were associated with the predefined behavioral states defined during the calibration, training, and testing protocol.

Two supervised ML classification models were trained using *scikit-learn*: (1) Support Vector Machine (SVM) with a linear kernel (C = 1), and (2) Random Forest (RF) classifier (n_estimators = 100, max_depth = 3, random_state = 42) (Ghimire and Lee, 2013; Munasinghe, 2018). SVMs were selected due to their effectiveness with small datasets and high-dimensional feature spaces, while RF models were chosen due to their robustness and feature importance analysis (Géron, 2019). Additionally, deep learning models were trained using *tensorflow*, leveraging a RTX 3060 GPU (6 GB VRAM) to train the static Artificial Neural Network (ANN), and the sequential Convolutional Neural Networks (CNN) and Recurrent Neural Networks (RNN), including Gated-Recurrent Unit (GRU) and Long-Short Term Memory (LSTM) cells (Géron, 2019). A systematic hyperparameter sweep as in Candela-Leal et al. (2022) evaluated number of neurons [2, 4, 8, 16], hidden layers [1, 2, 3], sequence length [5, 10, 20, 40, 80, 120], and random initialization seeds (n=10) , totaling 1,260 trained models. All architectures used the Adam optimizer (learning rate = 0.0005), a batch size of 128, categorical cross-entropy loss, and a final softmax layer. Static models used ReLU activations and ran up to 500 epochs ( < 1 s/epoch), whereas sequential models were trained up to 30 epochs ( ∼ 3 s/epoch): RNNs used *tanh* activations with *sigmoid* recurrent units, while CNNs used ReLU activations, 1×3 kernels, same padding, and 1D max-pooling (pool size = 2) between convolutional layers. These deep learning architectures feature automatic feature extraction and often yield high accuracy (Ramírez-Moreno et al., 2026), particularly in driver monitoring and facial analysis (Liu M. et al., 2021; Chen et al., 2021; Yu et al., 2024).

All architectures used the Adam optimizer (learning rate = 0.0005), a batch size of 128, categorical cross-entropy loss, and a final softmax layer. Static models incorporated ReLU activations and ran up to 500 epochs ( < 1 s/epoch). Sequential models were trained up to 30 epochs ( ∼ 3 s/epoch): RNNs used tanh activations with sigmoid recurrent units, while CNNs used ReLU activations, 1×3 kernels, same padding, and 1D max-pooling (pool size = 2) between convolutional layers.

Prior to analysis, the data went through several pre-processing steps: Frames without detected faces were removed ( ∼ 1.5%), timestamps were aligned to the start of the session to compute elapsed time, and a commanded state label was assigned according to the protocol. Blink rate per minute was recalculated offline considering non-causal 10-s rolling windows, and the training dataset was used to standardize itself and the testing dataset via *StandardScaler* for zero mean and unit variance.

To evaluate model’s performance on the training dataset, it was shuffled and split through a stratified k-fold cross-validation (k = 10), allowing model validation while preventing overfitting. To evaluate generalization, a model was trained on each training fold and was tested on a hold-out testing dataset collected from the same user under less controlled environmental conditions, which consisted in an outdoor environment where sun, noise, and wind were variant. This dataset allowed assessment of cross-session performance, simulating realistic deployment conditions where driver monitoring systems must operate at an exterior environment, where lighting and recording conditions might vary (Figures 1B,C).

The aforementioned model evaluation scheme was done for static models (SVM, RF, ANN), having 95% confidence intervals for each trained fold (n = 10) on both training and testing datasets. On the other hand, sequential models (RNN, CNN) cannot be trained in shuffled sampled distributions from stratified k-fold cross-validation, thus, they were trained on the whole training dataset while the testing dataset was used as a validation dataset for hyperparameter tuning, having 95% confidence intervals for each training random seed initialization (n = 10) on both training and testing datasets.

Classifier performance was evaluated using accuracy, f1-score, ROC-AUC, and confusion matrices. A confusion matrix summarizes correct and incorrect predictions (Chen, 2021) by reporting True Positives (TP), True Negatives (TN), False Positives (FP), and False Negatives (FN) (Table 2), and can be extended to multiclass classification problems (Géron, 2019). Accuracy was calculated as the proportion of correct predictions Equation 3a, while the f1-score Equation 3b, the harmonic mean of precision Equation 3c and recall Equation 3d, provides a more reliable measure for imbalanced datasets by favoring models with balanced precision and recall (Géron, 2019).

**TABLE 2:** Confusion matrix mapping true versus predicted labels. True Positive (TP), False Negative (FN), False Positive (FP), True Negative (TN).

		Predicted	
		Positive	Negative
True	Positive	TP	FN
	Negative	FP	TN


$$\text{Accuracy}=\frac{TP+TN}{TP+TN+FP+FN}$$ (3a)

$$\text{f}1-\text{score}=2\cdot \frac{\text{precision}\cdot \text{recall}}{\text{precision}+\text{recall}}$$ (3b)

$$\text{precision}=\frac{TP}{FP+TP}$$ (3c)

$$\text{recall }\left(TPR\right)=\frac{TP}{FN+TP}$$ (3d)

Moreover, Receiver Operating Characteristic (ROC) curves were generated by plotting the True Positive Rate (TPR) Equation 3d against the False Positive Rate (FPR) Equation 4, to evaluate the trade-off between recall and specificity (Chen, 2021). The corresponding Area Under the Curve (AUC) quantifies overall classifier performance, with higher values indicating a better model (Chen, 2021). To assess feature discriminability across multiple groups with unequal sample sizes, a Kruskal-Wallis H test was utilized to detect if there is a significant median difference for at least two of the groups (Boslaugh, 2012). Subsequent *post hoc* pairwise comparisons were adjusted using the Bonferroni correction to control the increase in Type I errors (FPR, Equation 4) when conducting multiple comparisons (Boslaugh, 2012).

$$\text{FPR}=\frac{FP}{TN+FP}$$ (4)

## 3 Results

### 3.1 Facial feature analysis per behavioral state

The facial monitoring system operated by tracking facial landmarks in real-time using the MediaPipe Face Mesh model to extract key behavioral features (Figure 3A). These included eye and mouth contours that were used to compute the EAR and MAR, as well as landmark points required for head pose estimation. During operation, the interface displayed real-time overlays of the detected facial mesh, alongside continuously updated values of EAR, MAR, and head rotation angles (Figure 3A); in addition to supporting facial landmarks recording and labeling for offline analysis. Under the implemented configuration, the facial tracking pipeline operated at approximately 16.66 frames per second at a resolution of 960 × 540 pixels, enabling reliable capture of facial metrics for subsequent calibration and behavioral state validation.

**FIGURE 3:**
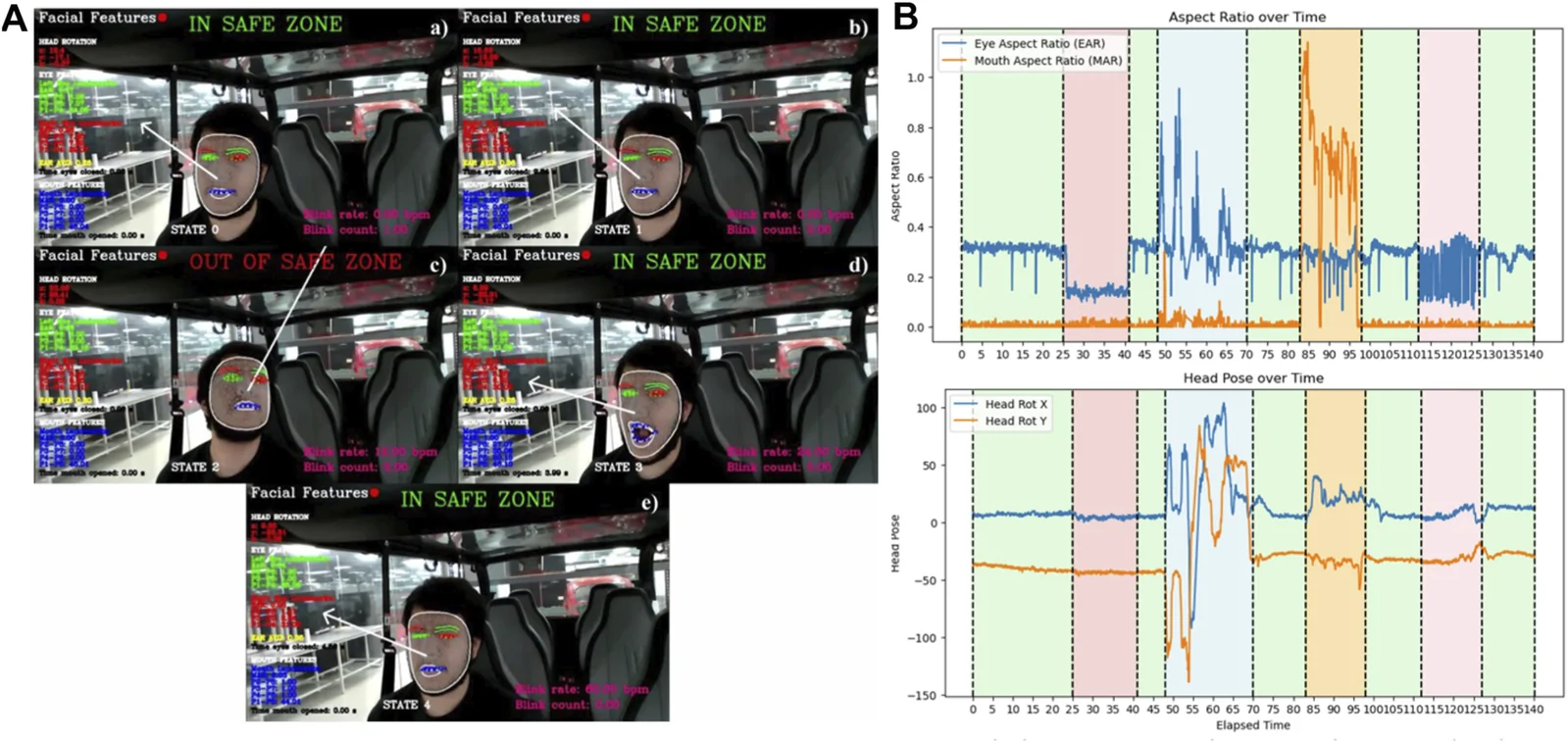
Facial features described behavioral states. **(A)** Validation demonstration with detected a) State 0 - Neutral, b) State 1 - Eyes closed, c) State 2 - Look outside safe zone, d) State 3 - Yawning, e) State 4 - Excessive blinking based on calibrated facial features thresholds. **(B)** EAR and MAR, and head rotation X/Y time series across the structured validation protocol, in which color green represents the State 0 while the alternating colors corresponds to the State 1, State 2, State 3, and State 4.

Following the previously mentioned training and testing protocol, two datasets were generated, consisting in a training dataset of 2,332 processed frames and a testing dataset of 2,257 processed frames. Each entry corresponded to a single video frame and included timestamped measurements of the following variables: EAR (left and right), MAR, head rotation angles (X,Y), face detection status, blink rate. Overall, the face detection success rate reached ∼ 98.5%, demonstrating reliable facial landmark tracking throughout the recording session.

Time-series analysis was performed to observe how biometric facial indicators varied across behavioral states (Figure 3B). At each state, distinct patterns were observed, for instance: Reduced EAR values were strongly associated with prolonged eye closure in State 1, while elevated MAR values corresponded to yawning in State 3. Head rotation angles showed clear deviations during State 2 when the user was instructed to look outside the calibrated gaze zone. Finally, State 4 was characterized by a significantly increased blink rate compared to the neutral condition.

Moreover, a more nuanced raw feature space representation with the states is shown at Figure 4A, in which calibration and threshold values are overlaid with dotted lines according to Table 1. Each point represents a frame, and it can be seen that the thresholds that support the threshold-based finite-state model are able to overall separate states by considering key discriminative features such as the aforementioned state-feature relationships, in which state-feature relationships were consistent across the training and testing datasets, collected in a controlled indoor environment, and in a less controlled outdoor environment, respectively.

**FIGURE 4:**
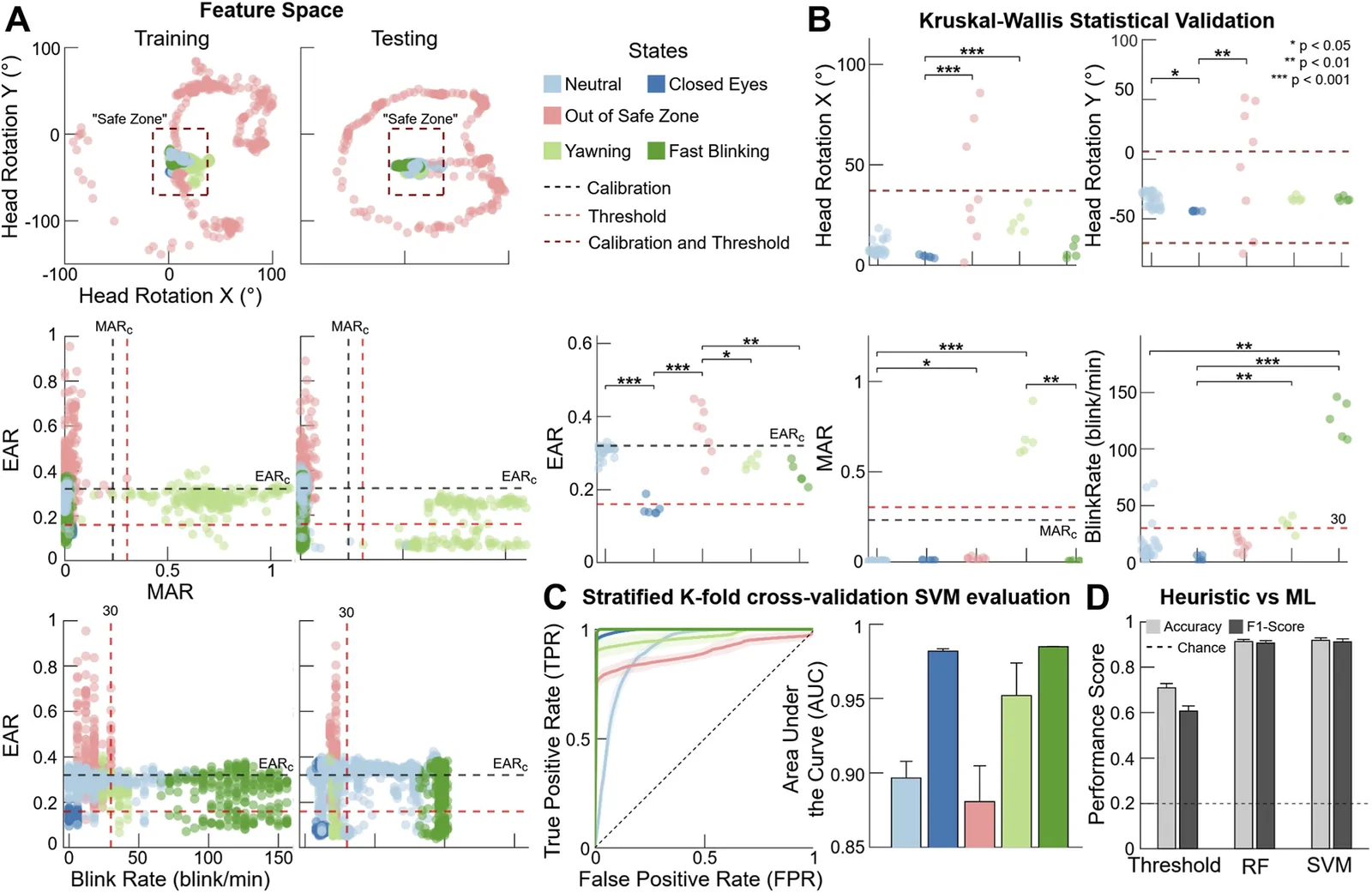
Facial features were state-specific. **(A)** Raw training and testing dataset facial features (head rotation X/Y, EAR, MAR, and blink rate) color-coded by state (neutral, closed eyes, out of safe zone, yawning, fast blinking), with dotted lines indicating calibration/threshold values according to Table 1. **(B)** Kruskal-Wallis statistical test results comparing features across states, with significance denoted by asterisks (*). **(C)** Receiver Operating Characteristic (ROC) curves per state generated via stratified k-fold cross-validation of a linear Support Vector Machine (SVM) model on the training dataset. **(D)** Stratified k-fold cross-validation performance comparison between the heuristic, Random Forest (RF), and SVM models, with a dotted line indicating the 20% chance level.

To assess feature discriminability across states, the training dataset was downsampled on the neutral state and features were averaged over 2.5-s non-overlapping windows (42 frames) to increase samples independence. Kruskal-Wallis tests followed by Bonferroni-corrected *post hoc* comparisons revealed significant differences for all features ( p<0.001 , Figure 4B). For head rotation X, the closed eyes state differed significantly from out of safe zone and yawning (p<0.001) , while for head rotation Y, closed eyes differed from neutral (p<0.05) and out of safe zone (p<0.01) . EAR and MAR successfully isolated their objective states: for EAR, closed eyes differed from neutral and out of safe zone (p<0.001) , while out of safe zone differed from yawning (p<0.05) and fast blinking (p<0.01) ; for MAR, yawning differed from neutral (p<0.001) and fast blinking (p<0.01) , while out of safe zone differed from neutral (p<0.05) . Finally, fast blinking frames consistently exceeded the blink rate threshold despite some false positives from neutral and yawning; fast blinking differed significantly from neutral (p<0.01) and closed eyes (p<0.001) , while yawning differed from closed eyes (p<0.01) .

### 3.2 Machine learning driver state classification

Automatic classification of driver states was evaluated by training both SVM and RF classifiers using the training facial features (EAR, MAR, head rotation, blink rate). A stratified k-fold cross-validation scheme (k = 10) was applied to evaluate model variance, assess state-dependent accuracy across runs, and compute 95% confidence intervals for the performance metrics. Using the SVM model’s probability scores via its One-vs-All configuration (Géron, 2019), ROC curves through TPR and FPR, and average AUC values were calculated for each class (Figure 4C). The highest performance was achieved for closed eyes and fast blinking (98% AUC), followed by yawning (95%), out of safe zone (88%), and neutral states (89%). Across the folds, the SVM and RF models demonstrated comparable performance: SVM achieved 91% accuracy and 91% F1-score, while RF achieved 92% accuracy and 91% F1-score, outperforming the baseline heuristic model (Figure 4D). This threshold-based, finite-state model (Table 1) was evaluated on the unshuffled testing segments to preserve temporal dynamics, and yielded a modest 71% accuracy and 61% F1-score, though it remained well above the 20% chance level.

Furthermore, on the unseen testing dataset, the SVM achieved 90% accuracy and 89% f1-score, demonstrating strong generalization performance and being higher than heuristic F1-score (81%) (Figure 5C). The confusion matrix (Figure 5D) indicated high precision and recall across most behavioral states, with excellent performance for neutral (95%), closed eyes (96%), yawning (93%), and fast blinking (94%). While major misclassifications occurred in out of safe zone (66%) with neutral (31%), likely due to overlapping head rotation angles close to the defined safe-zone limits. On the other hand, the RF model achieved 82% accuracy and 70% f1-score in the testing dataset (Figure 5C). Interestingly, its confusion matrix at Figure 5D is different, as the model correctly detected states such as neutral (99%), yawning (93%), and fast blinking (91%), with major misclassifications at closed eyes (47%) and out of safe zone (67%) with neutral (53%, 37%).

**FIGURE 5:**
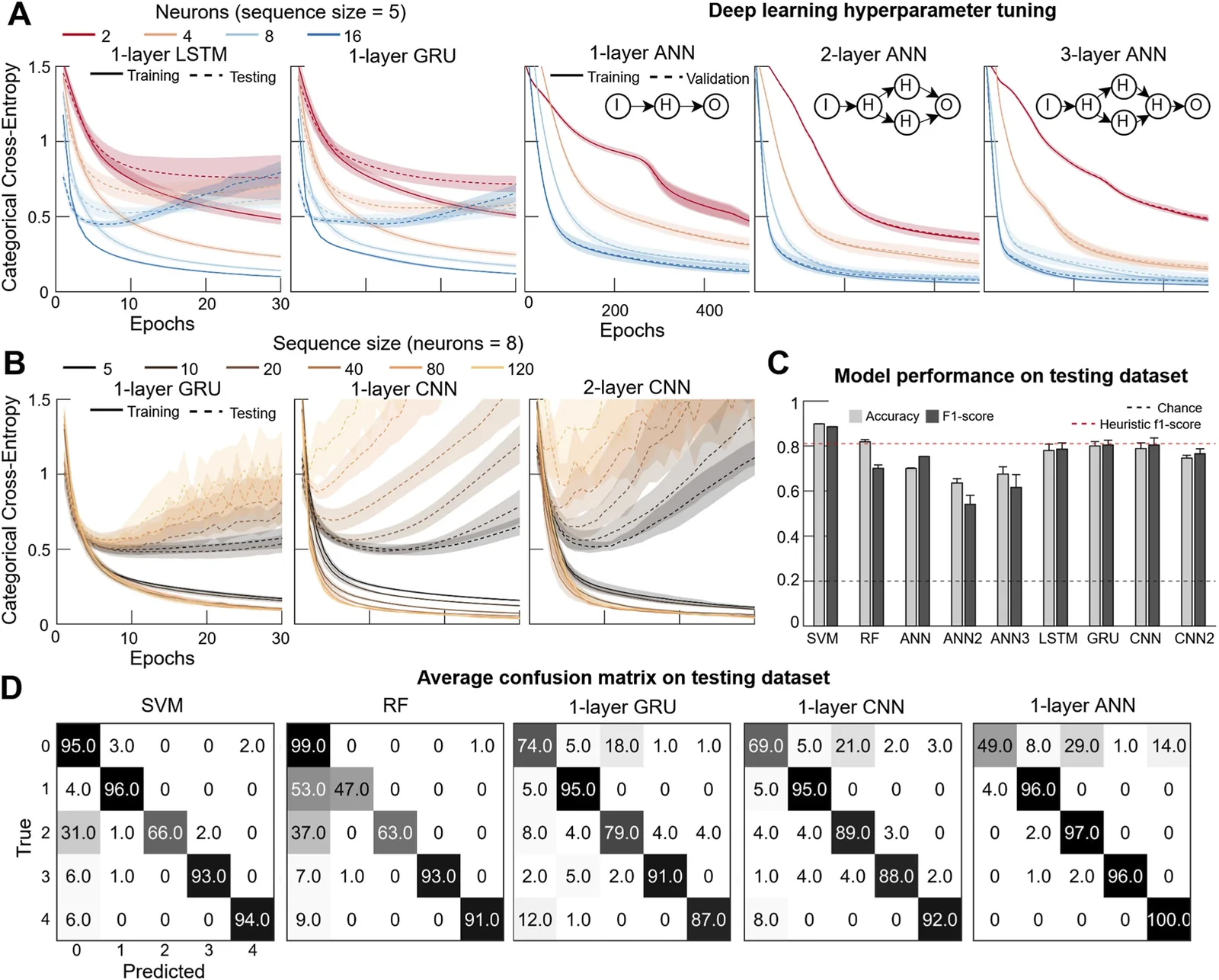
Cross-session model evaluation and hyperparameter tuning. **(A,B)** Hyperparameter optimization showing categorical cross-entropy loss across **(A)** neurons ([2, 4, 8, 16], sequence length = 5) and **(B)** sequence lengths ([5, 10, 20, 40, 80, 120], neurons = 8). Sequence models (LSTM, GRU, CNN) reflect 95% CI variability across random training seeds (n = 10) with the testing dataset, while static models (ANN) reflect 95% CI variability across 10-fold stratified cross-validation. **(C)** F1-scores on the test set across static (SVM, RF, ANN; CI across cross-validation folds) and sequence (LSTM, GRU, CNN; CI across random seeds) architectures relative to chance (dotted black line) and the heuristic model (dotted red line). **(D)** Average test set confusion matrices across trained folds (static) and seeds (sequence), considering the behavioral states at Table 1 (0: Neutral, 1: Closed Eyes, 2: Out of Safe Zone, 3: Yawning, 4: Fast Blinking). Artificial Neural Network (ANN), Convolutional Neural Network (CNN), Gated Recurrent Unit (GRU), Long Short-Term Memory (LSTM), Random Forest (RF), Support Vector Machine (SVM).

### 3.3 Deep learning driver state classification

In contrast to classical ML models, deep learning architectures require extensive hyperparameter tuning to prevent underfitting and overfitting. Testing 1D-GRU and 1D-LSTM models across number of neurons [2, 4, 8, 16] with a fixed sequence size of 5 showed that 2 and 4 neurons underfitted, 16 overfitted, and LSTM exhibited higher variance across seeds than GRU (Figure 5A). Testing the same number of neurons across 1D-, 2D-, and 3D-ANN architectures similarly revealed underfitting at 2 and 4 neurons, particularly in 1D and 3D, as well as performance stabilization after 8 neurons. Hence, 8 neurons was selected as the optimal number of neurons for both static and sequential deep learning models (Figure 5A).

Afterwards, having a fixed number of neurons, increasing sequence size increased training overfitting across all GRU and CNN architectures, reducing test performance. Overfitting was least sensitive to sequence size in 1D-GRU, moderately sensitive in 2D-CNN, and most sensitive in 1D-CNN. However, at the optimal sequence of 5, 1D-CNN exhibited less total overfitting than 2D-CNN despite its higher rate of change (Figure 5B).

On overall deep learning test performance (Figure 5C), static models (SVM, RF, ANN) were evaluated on unseen data with cross-validation confidence intervals, while sequential models (LSTM, GRU, CNN) used test data during hyperparameter tuning, with confidence intervals derived from seed initializations. ANNs performed worst (2D: 63% accuracy/54% F1-score; 3D: 67%/61%; 1D: 70%/75%), followed by 2D-CNN (74%/76%) and LSTM (78%/78%). GRU (80%/80%) and 1D-CNN (78%/80%) performed best among deep learning architectures, though none exceeded the heuristic baseline F1-score.

Finally, an average confusion matrix compared the best 1-layer GRU, CNN, and ANN architectures. All three performed excellent at detecting eyes closed (95%–96%). GRU and CNN showed similar accuracy for yawning (91%, 88%) and fast blinking (87%, 92%), but diverge on neutral and out of safe zone states: GRU achieved better balance (79% out of safe zone, 74% neutral), while CNN favored non-neutral states (89% out of safe zone, 69% neutral), yielding more false positives. On the other hand, ANN heavily biased toward non-neutral predictions, performing well on out of safe zone (97%), yawning (96%), and fast blinking (100%), but failing on neutral (49%) due to severe misclassification into out of safe zone (29%) and fast blinking (14%).

## 4 Discussion

This study explored two complementary components of human-vehicle interaction in autonomous systems: System transparency through a Human Machine Interface (HMI) and behavioral awareness through a facial feature-based Driver Monitoring System (DMS); thus, showing the potential of safe and effective deployment of autonomous vehicles with mechanisms that both communicate the vehicle’s internal state to the user and monitor the user’s behavioral condition (Wilson et al., 2022). The results demonstrate that these two perspectives can be implemented within a unified experimental platform, as the HMI successfully integrated vehicle telemetry, localization, obstacle detection, and system status into a real-time visualization interface, enabling clear communication of operational states during autonomous navigation (Figure 1). Although these communication features were qualitative, the experiments indicate that such interfaces can provide meaningful situational real-time feedback to users, supporting the broader goal of improving transparency and trust in autonomous vehicle behavior (Ekman et al., 2018).

Specifically, in the facial feature-based DMS (Figure 2), the extracted biometric features were interpretable and consistent across sessions (Figure 4). These patterns suggest that features such as Eye Aspect Ratio (EAR), Mouth Aspect Ratio (MAR), and head pose provide meaningful indicators of driver behavioral states, enabling users to understand how the system reaches its predictions. This transparency is important for building trust and supporting interpretable models suitable for real-world deployment (García-Hernández et al., 2024; Wang et al., 2022). In contrast, many deep learning approaches for driver’s cognitive and behavioral state recognition, such as CNNs and RNNs, can achieve high accuracy and benefit from automatic feature extraction (Ramírez-Moreno et al., 2026), but they typically require large datasets and operate as black-box models with limited explainability (Espino-Salinas et al., 2024; Li et al., 2020; Zhu et al., 2021; Xiao et al., 2022; Zohaib et al., 2024). Although Explainable Artificial Intelligence (XAI) techniques, such as Grad-CAM, can provide partial interpretability (Gebele et al., 2024), deployed models based on full-face representations (e.g., DeepFace (Taigman et al., 2014)) may bias predictions toward neutral expressions, suffer from limited generalization, and often fail to communicate the reasoning behind predictions (Olivas et al., 2021). By contrast, the proposed approach relies on explicit facial metrics that directly reflect behavioral cues, enabling both interpretable classification and continuous monitoring. Although the models were trained and tested offline, the lightweight feature set and classifiers suggest that the system could be implemented in real-time with visual feedback through the face mesh interface (Davoli et al., 2020; Zhu et al., 2021), providing a foundation for future driver monitoring systems.

The facial feature-based monitoring framework demonstrated the feasibility of detecting driver behavioral states using non-intrusive computer vision techniques (Figure 3), an important requirement for deployment in consumer vehicles (Mehmood et al., 2024; Prabhakar and Biswas, 2021; Ramírez-Moreno et al., 2026). The landmark-based features were state-specific (Figures 4A,B), as they demonstrated high discriminability across states through a One-vs-All SVM model (AUC ≥ 88%) (Figure 4C), and could differentiate between states through classical ML, as SVM and RF models had high performance on stratified k-fold cross validation ( ∼ 91% accuracy/ ∼ 91% F1-score), significantly outperforming the threshold-based finite-state model used during recording (71%/58%) (Figure 4D).

On the hold-out testing dataset, the SVM classifier achieved a similar cross-validation performance (90%/89%), while the RF classifier failed to generalize (82%/70%) and performed worse than the threshold-based model (85%/81%) (Figure 5C). Furthermore, confusion matrices revealed distinct algorithmic behaviors (Figure 5D), as the SVM model primarily confused the out of safe zone (66%) with the neutral (31%) state, while the RF model not only showed confusion for these states (63% with 37%), but also struggled heavily with closed eyes (47% with 53%). This discrepancy can be attributed to differences in how each algorithm handles ambiguous feature boundaries: (1) while the EAR was highly discriminant for closed eyes with neutral state (p<0.001) , it was also statistically significant for the out of safe zone state (p<0.001) ; (2) conversely, head rotation angles were significant for the out of safe zone state with closed eyes (X-axis p<0.001 ; Y-axis p<0.01 ), but not with neutral state (X-axis p>0.05 ; Y-axis p>0.05 ) (Figures 4A,B). On the confusion matrix, regarding SVM and RF, with out of safe zone and closed eyes misclassification, the errors tended to predict the neutral class due to overlapping boundaries on multi-state features with class imbalance (Kim and Kim, 2020), as SVM margin maximization absorbs overlap into majority class due to the majority class samples dominate the overlapped region, disregarding the minority class samples (Mahmood et al., 2025), while RF majority voting structure defaults to the dominant class when trees encounter ambiguous, overlapping instances (Bader-El-Den et al., 2019). Specifically, RF failed to accurately classify the closed eyes state despite the highly discriminative EAR feature with the neutral state (p<0.001) . This highlights a key limitation of RF models across overlapping feature distributions, as hyperparameter tuning, particularly for maximum depth, is highly sensitive, in which high depth increases model complexity and leads to overfitting (Munasinghe, 2018), while low depth restricts generalization across complex feature distributions; thus, even though the RF model performed well under controlled conditions, larger datasets or ensemble strategies may be necessary for robust real-world deployment (Heidari et al., 2025).

Beyond the classical ML comparison, we further evaluated whether deep learning architectures could better resolve this state ambiguity, by training static (ANN) and sequential (RNN, CNN) deep learning models on the calculated features. Hyperparameters tuning prevented underfitting and overfitting through optimal configurations: 8 neurons (Figure 5A), a sequence size of 5 for sequence models (Figure 5B), and 1 hidden layer (Figures 5A,B), as larger values increased overfitting. None outperformed the SVM model (90%/89%) or the threshold-based model (85%/81%). Sequential models performed best with GRU (80%/80%) and CNN (78%/80%), whereas static deep learning had the highest performance with ANN (70%/75%). Interestingly, classical ML models (SVM, RF) primarily misclassified out of safe zone instances as neutral, whereas deep learning models showed the opposite pattern, misclassifying neutral instances as out of safe zone (Figure 5D). While SVM and RF defaulted to majority-class predictions in overlapping feature spaces might be due to margin maximization (Mahmood et al., 2025), majority voting (Bader-El-Den et al., 2019), or lack of temporal context (Sheykhmousa et al., 2020), here deep learning architectures shifted predictions toward minority classes. This shift was not strictly sequence-dependent, as ANN exhibited a similar bias; however, GRU and CNN maintained a much better class balance, whereas ANN heavily biased toward non-neutral states (49% neutral accuracy) (Figure 5D). Overall, deep architectures transform the input space to map overlapping regions into separable cluster representations (Mahmood et al., 2025) that implicitly reduce overlap (Ghosh et al., 2022). This feature-space transformation creates denser, highly generalizable clusters for frequent classes, enhancing discrimination of non-neutral patterns (Kim and Kim, 2020) and capturing minority-class characteristics more effectively than traditional classifiers (Chen et al., 2024). Moreover, regarding inference efficiency, GPU-accelerated deep learning models required ∼ 55 ms per prediction (RTX 3060 6 GB), compared to CPU-based inference for RF ( ∼ 5 ms) and SVM ( ∼ 0.2 ms). Although all models met the 60 ms real-time processing window ( $f_{s}=16.67$ Hz), SVM was the most efficient choice after accounting for additional pipeline latencies (transmission latency, landmark extraction, pre-processing).

Given multi-state feature ambiguity, SVM demonstrated higher stability and generalization across datasets, as its errors were mainly at the out of safe zone state, which did not have strong feature definitions with the neutral state (p>0.05) . Therefore, for multi-class behavioral classification tasks derived from facial metrics, SVM is preferred: its marginalized, linear decision boundary aligns consistently with the structured and low-dimensional feature space distribution and discriminability (Cervantes et al., 2020), facilitating easier implementation, faster inference efficiency, better interpretability, and higher trust (Liu X. et al., 2021).

### 4.1 Limitations

Despite the promising behavioral state recognition results, several limitations should be considered. First, the small sample size (N = 1) in a stationary vehicle, not being able to test model generalization across diverse facial morphologies, ages, and dynamic driving conditions (e.g., variable lighting and multi-passenger occupancy); however, the developed calibration and structured training protocol, supported by a real-time threshold system, ensured high-quality data collection and a personalized model that successfully reduced cross-session degradation. Utilizing an interior camera provided structural stability, and enabled reliable face landmark tracking with consistent camera positioning relative to the driver across sessions. Although further validation is needed to explore more diverse in-cabin and external environments, this proof-of-concept demonstrates the feasibility of behavior classification using non-intrusive and interpretable facial features at an autonomous vehicle. Future studies should include a larger and diverse cohort while leveraging the structured calibration protocol and heuristic model to ensure reliable, personalized behavioral monitoring; thus, having a cohesive, universal machine learning model with generalization ability across different ages, facial features, and environment conditions.

An important limitation was the confusion between the out of safe zone and neutral states across machine learning and deep learning models, driven by transitional head rotations during gaze shifts that overlapped with the neutral state’s feature range. Future protocols could refine labeling via real-time human-in-the-loop correction or pre-trained models for automated frame segmentation. Alternatively, state disambiguation could be directly addressed using Electrooculography (EOG) (Picot et al., 2009; Bafna and Hansen, 2021) or eye-gaze features (Bafna and Hansen, 2021; Ghosh et al., 2024), though the former is intrusive for the driver and the latter requires high-resolution optics or dedicated close-up cameras (Aunsri and Rattarom, 2022; Xia and Ruan, 2007; Roman et al., 2025). Further optimization of the calibration procedure could also determine the minimum recording time required to achieve reliable predictions while reducing user setup time (Ramírez-Moreno et al., 2021). Finally, integrating additional multi-modal physiological data (e.g., EDA, EOG, or EEG) could help validate the facial feature-based indicators and improve robustness and inter-subject generalization (Laurent et al., 2013).

## 5 Conclusion

Altogether, these findings suggest that combining transparent system communication with user-state monitoring may represent a promising direction for human-centered autonomous vehicle design, where both the vehicle and the user contribute to maintaining safe interaction. This framework also supports future deployment in shared mobility contexts, with potential extensions including multi-modal user feedback, real-time driver alerting mechanisms, and multi-user calibration strategies for broader adoption.

The ML analysis provided valuable insights for developing stable and deployable behavioral monitoring models. The observed differences in model performance highlight the importance of not only clearly defining behavioral states but also selecting algorithms that generalize well beyond the training data. While the SVM model showed strong performance overall, the reduced accuracy in the out of safe zone state may reflect transitional dynamics in the protocol and overlapping feature representations with the neutral state. In contrast, the different performance of the RF model across the out of safe zone, closed eyes, and neutral state, suggests overfitting to session-specific patterns in the training data; hence, reducing cross-session performance. These findings highlight the importance of model selection, hyperparameter tuning, and dataset size when deploying DMS. In particular, linear SVM models appear well suited for classification tasks based on low-dimensional, non-intrusive facial features, where interpretability, cross-session performance, inference efficiency, and limited training data are key considerations. Ultimately, these results emphasize that effective driver monitoring systems require not only accurate behavioral indicators but also modeling approaches that remain robust at deployment.

## Data Availability

The raw data supporting the conclusions of this article will be made available by the authors, without undue reservation. Code is available in GitHub: https://github.com/miltoncandela/facial-behavior-dms.
